# Incidence, demographic characteristics, and geographic distribution of sickle cell trait and sickle cell anemia births in Michigan, 1997–2014

**DOI:** 10.1002/mgg3.795

**Published:** 2019-06-17

**Authors:** Sarah L. Reeves, Hannah K. Jary, Jennifer P. Gondhi, Mary Kleyn, Kayte Spector‐Bagdady, Kevin J. Dombkowski

**Affiliations:** ^1^ Department of Pediatrics, Susan B. Meister Child Health Evaluation and Research Center University of Michigan Ann Arbor Michigan; ^2^ Michigan Department of Health and Human Services Lansing Michigan; ^3^ Department of Obstetrics and Gynecology, Center for Bioethics & Social Sciences in Medicine University of Michigan Ann Arbor Michigan

**Keywords:** Michigan, newborn screening, sickle cell anemia, sickle cell trait

## Abstract

**Background:**

This study describes the incidence, demographic characteristics, and geographic distribution of sickle cell anemia (SCA) and sickle cell trait births in Michigan.

**Methods:**

Michigan newborn screening records and birth certificates (1997–2014) were used to identify sickle cell trait and SCA births, as well as demographic characteristics and mother's residential address. Incidence was calculated overall and by county.

**Results:**

During the study period, there were 592 SCA births and 33,404 sickle cell trait births in Michigan. The majority of SCA (86.3%) and trait (80.2%) cases were among children who were black. Children with SCA were born in 23% of Michigan counties; children with trait were born in 93%.

**Conclusion:**

Compared to SCA, sickle cell trait births occur at 50‐fold greater incidence and have a substantially expanded geographic distribution. Further research is necessary to understand the most appropriate and impactful use of resources to increase the proportion of families and adults that are aware of their sickle cell trait status.

## 1 INTRODUCTION

Sickle cell disease is an autosomal recessive genetic red blood cell disorder predominately affecting minority populations in the US (Arhin, 2019; Hassell, 2010). Two genes for sickle hemoglobin are inherited from the parents of those that are affected. Among those with sickle cell disease, there are many subtypes, including hemoglobin (Hb)SS, HbSC, HbS beta^0^ thalassemia, HbSD, HbSE, and HbSO. HbSS and HbS beta^0^ thalassemia present in a similar clinical manner and are commonly referred to together as sickle cell anemia (SCA), which is the most severe subtype of sickle cell disease (2017b; National Heart Lung & Blood Institute, 2014; Stuart & Nagel, 2004). SCA is associated with substantial morbidity and affects 1 in every 600 births of black children in the US (Hassell, 2010). The most common complication of SCA is pain, which is also the main cause of emergency department visits and hospitalizations for people with SCA (CDC, 2017a). Other significant complications include anemia, stroke, infection, hand‐foot syndrome, acute chest syndrome, splenic sequestration, vision loss, leg ulcers, deep vein thrombosis, and pulmonary embolism (CDC, 2017a).

Approximately 3 million Americans have sickle cell trait (HbAS), which is the gene carrier status of sickle cell disease (2017b; Taylor, Kavanagh, & Zuckerman, 2014). Those with sickle cell trait have inherited only one gene for sickle hemoglobin from a parent; the other hemoglobin gene is normal. Although generally asymptomatic, sickle cell trait has been linked to rare health problems (2017b; Kato et al., 2018; Naik et al., 2014, 2017, 2018; Nelson et al., 2016; Piel, Steinberg, & Rees, 2017; Tsaras, Owusu‐Ansah, Boateng, & Amoateng‐Adjepong, 2009). For example, sickle cell trait has been linked to an increased risk of renal complications and splenic infarct; these complications are often associated with extreme environments, particularly in situations of increased atmospheric pressure, low oxygen levels, dehydration, and high altitudes (Brousse, Buffet, & Rees, 2014; CDC, 2017; 2019; Naik et al., 2014). The vast majority of those with sickle cell trait do not exhibit any signs or symptoms associated with SCA; however, those with sickle cell trait may face challenges related to making informed family planning decisions given its heritability pattern (CDC, 2017; 2017b; Gallo et al., 2010).

All states perform newborn screening (NBS) at birth to screen for genetic conditions for which early diagnosis and treatment can have life‐saving benefit (Kavanagh, Wang, Therrell, Sprinz, & Bauchner, 2008). All newborns are screened for all hemoglobin variants, including sickle cell trait and SCA. However, significant variation exists in follow‐up policies across states and between disease subtypes (SCA vs. sickle cell trait), and few policies ensure communication of positive sickle cell trait results to families and physicians (Kavanagh et al., 2008). For example, in Michigan, families with a child born with sickle cell trait are proactively contacted by the Sickle Cell Disease Association of America—Michigan Chapter to provide genetic educational resources (Smith, Lyon‐Callo, & Young, 2015). In Maryland, a letter is sent to the pediatrician of any child with a positive sickle cell trait NBS result; however, no standardized follow‐up procedures are in place for families (Maryland Department of Health, [Link]).

Given the differences in follow‐up and prevalence between SCA and sickle cell trait, our objective was to use NBS records to describe the incidence, demographic characteristics, and geographic distribution of SCA and sickle cell trait births in Michigan.

## 2 MATERIALS AND METHODS

### 2.1 Study population

Our study population consisted of children with SCA (specifically, HbSS, and Hb beta^0^ thalassemia) and sickle cell trait born in the state of Michigan from 1997 to 2014. NBS records were used to identify individuals with these hemoglobinopathy variants. NBS records reflect hemoglobinopathy results that were obtained using methods such as high performance liquid chromatography and isoelectric focusing (Michigan Department of Health & Human Services Newborn Screening Program, 2015). After referral to a pediatric hematologist, hemoglobin electrophoresis is performed for newborns with hemoglobinopathy results suggestive of disease. Therefore, the NBS records reflective of the results of these tests are the gold standard of identification of children with sickle cell trait and SCA. Children with sickle cell trait or SCA were then linked to Michigan Medicaid records with electronic birth certificates using a previously validated method (Korzeniewski et al., 2010; Reeves et al., 2014). Sex of the child and mother's address at time of birth were obtained from birth certificates, while race/ethnicity of the child was obtained from NBS records. Race/ethnicity of the child is determined in NBS records as a combination of the self‐identified race/ethnicities of both parents, as recorded by hospital staff at the time of birth (Michigan Department of Health & Human Services, 2019).

### 2.2 Statistical analysis

#### 2.2.1 Overall incidence

The incidence of SCA and sickle cell trait was calculated as the number of SCA or sickle cell trait births divided by the number of live births, both from 1997 to 2014 overall, and by each year. Number of live births to Michigan residents by year were obtained from Vital Records at the Michigan Department of Health and Human Services (MDHHS) (Radford, 2017).

#### 2.2.2 Child demographic characteristics

Race/ethnicity and sex were summarized across the study period for all SCA and sickle cell trait births.

#### 2.2.3 Geographic distribution of cases

ArcGIS 10.1 was used to geocode the mother's residential address at time of birth to the county level for all SCA and sickle cell trait births. Using Vital Records from MDHHS, the incidence of SCA and sickle cell trait at the county level were calculated as the number of SCA and sickle cell trait births from 1997 to 2014 divided by the number of live births in the same time frame within each county. The incidence rates within each county were calculated per 10,000 live births and used to create county‐level choropleth maps. Across the study period, the proportion of SCA and sickle cell trait births within the City of Detroit was also calculated, as Detroit is home to the highest percentage of black residents in any US city (United States Census Bureau, 2011). Detroit is also the largest and most populous city in Michigan and the most racially segregated large metropolitan area in the United States (Darden, Rahbar, Jezierski, Li, & Velie, 2010; United States Census Bureau, 2012).

## 3 RESULTS

### 3.1 Overall incidence

Between 1997 and 2014, 592 children were born in Michigan with SCA among a total of approximately 2.2 million births, for an incidence rate of 2.6 per 10,000 live births (Michigan Department of Health & Human Services, 2017; Radford, 2017). Within this same time frame, 33,404 children were born with sickle cell trait, for an incidence of 148.6 per 10,000 live births.

### 3.2 Child demographic characteristics

The majority of children with SCA and sickle cell trait were black (86.3% and 80.2%, respectively). Among SCA births, 2.5% were white, 1.2% were multiracial, and 8.3% were of unknown race. Among sickle cell trait births, 7.0% were white, 5.3% were multiracial, and 5.9% had unknown race. The relative distribution of race for each hemoglobin status remained relatively constant over time. The distribution of sex was approximately equal for both SCA and sickle cell trait (Table 1).

**Table 1:** Distribution of race/ethnicity and sex of child by hemoglobin status, Michigan 1997‐2014, *n* (%)

	Sickle cell anemia *n* = 592	Sickle cell trait *n* = 33,404
Race		
White	15 (2.53)	2,345 (7.02)
Black	511 (86.32)	26,791 (80.20)
American Indian	<5	233 (0.70)
Asian/Pacific Islander	<5	74 (0.22)
Arab Descent	8 (1.35)	248 (0.74)
Multiracial	7 (1.18)	1,753 (5.25)
Unknown	49 (8.28)	1960 (5.87)
Ethnicity		
Hispanic	7 (1.18)	686 (2.05)
Sex		
Female	296 (50.00)	16,215 (48.54)
Male	285 (48.14)	16,964 (50.78)
Unknown	11 (1.86)	225 (0.67)

### 3.3 Geographic distribution of cases

**Figure 1:**
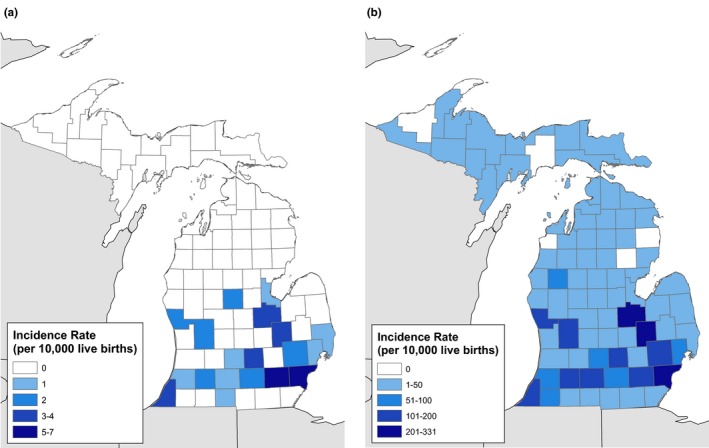
Incidence rates of sickle cell anemia (a) and sickle cell trait (b) births in Michigan, 1997‐2014

Geocoding was successful for over 95% of SCA and sickle cell trait births. SCA births occurred in 22.9% of counties (19 of 83) (Figure 1). A total of 49.0% (*n* = 290) of all SCA births occurred with the mother's residential city of Detroit. During this time period, there were 233,837 births in the city of Detroit; the incidence of SCA was 12.4 per 10,000 births in the city.

Sickle cell trait births occurred in all but 6 of Michigan counties (92.8%, Figure 1). A total of 40.7% (*n* = 13,595) of all sickle cell trait births occurred with the mother's residential city of Detroit, for an incidence of 581.4 per 10,000 births in the city. The counties with zero SCA and sickle cell trait births were located in rural regions of the Upper Peninsula and northern Michigan, which are populated mostly by white residents.

## 4 DISCUSSION

This is the first study to assess differences in the geographic distribution of SCA and sickle cell trait cases. Our assessment of statewide NBS results indicated that sickle cell trait cases in Michigan are much more geographically dispersed than SCA cases. Consistent with other studies, sickle cell trait occurred over 50 times more frequently than SCA (Pass et al., 2000; Peces & Peces, 2011). These results emphasize that outreach to individuals and families impacted by sickle cell trait should be offered throughout the state and not limited to areas of high sickle cell disease prevalence.

As SCA is an autosomal recessive genetic disorder, knowledge of sickle cell trait status, as well as the implications of this status are integral to making informed family planning decisions (Gallo et al., 2010; Taylor et al., 2014). Given the lack of guidelines for information dissemination to individuals with sickle cell trait, the relatively benign nature of the condition, and the age at which people are screened for sickle cell trait, it is not surprising that many adults living with sickle cell trait are unaware of their status (Arhin, 2019; Treadwell, McClough, & Vichinsky, 2006). One study found that only 16% of adults of reproductive age with sickle cell trait were aware of their own status (Treadwell et al., 2006). Unfortunately, there is little evidence to guide a best practice for increasing the proportion of families, adolescents, and adults that are aware of their sickle cell trait status.

One potential strategy to increase the proportion of aware families is utilizing NBS program outreach and primary care engagement to inform families of their newborn's sickle cell trait status. For SCA births, follow‐up within state health departments generally includes referral to pediatric hematology/oncology for disease education and penicillin prophylaxis (Michigan Department of Health & Human Services Newborn Screening Program, 2015). In contrast, although all sickle cell trait births are also identified through the NBS process, they involve significantly less follow‐up with key stakeholders such as families, physicians, and hospitals. In some states, no key stakeholders are even alerted of a positive NBS result for sickle cell trait (Kavanagh et al., 2008). Improved communication to families through NBS programs could be directly provided through the allocation of additional public health resources. For example, a study of all US states indicated that for each additional NBS follow‐up staff member, there was an 8.5% increase in the number of stakeholders that were notified of the child's sickle cell trait status (Kavanagh et al., 2008).

Primary care engagement may be another method to increase the proportion of families aware of their child's sickle cell trait status. NBS programs could engage primary care providers through direct, electronic delivery of sickle cell trait screening results into the electronic health record (Pecker & Naik, 2018; Taylor et al., 2014). Primary care providers, such as physicians and nurses, could then play a larger role in the effective reporting, patient education, and follow‐up of those who screen positive for sickle cell trait (Arhin, 2019). Yet, it is important to note that evidence is lacking regarding the long‐term outcomes of outreach efforts to parents on the knowledge of sickle cell trait status among adolescents and adults. However, information regarding an infant's sickle cell trait status may potentially inform the parents’ family planning decisions moving forward. Therefore, additional research should be conducted to identify the most effective strategies to inform families of their newborn's sickle cell trait status.

For adults who are unaware of their sickle cell trait status, genetic screening at reproductive age may be a consideration. In the United States, pre and postconception genetic screening for those of African, Southeast Asian, and Mediterranean heritage is recommended by the American College of Obstetrics and Gynecology, due to the higher risk of these individuals being carriers of hemoglobinopathies (The American College, 2007). However, survey data of obstetrics and gynecology fellows show that screening practices were not consistently conducted, and general knowledge of sickle cell trait and sickle cell disease screening was low (Azonobi, Anderson, Byams, Grant, & Schulkin, 2014). Additional benefits to screening adolescents and adults revolve around potential health‐related impacts of sickle cell trait, particularly as the risk of rare complications of sickle cell trait may increase across the lifespan (Kato et al., 2018; Naik et al., 2014, 2017, 2018; Nelson et al., 2016; Piel et al., 2017; Tsaras et al., 2009). These morbidities may include stroke, spleen and renal complications, and risks associated with extreme conditions, such as vigorous exercise and high altitudes (CDC, 2017; Kato et al., 2018; Naik et al., 2014, 2017, 2018; Nelson et al., 2016; Piel et al., 2017; Tsaras et al., 2009). For example, the NCAA requires all athletes to either be tested or sign a waiver of liability due to these risks, although this requirement has been controversial (Aloe, Krishnamurti, & Kladny, 2011; American Society of Hematology, 2012; Eichner, 2010; Ferrari, Parker, Grubs, & Krishnamurti, 2015; Jordan et al., 2011; Koopmans & Ross, 2012; Tarini, Brooks, & Bundy, 2012).

Another potential strategy to increase the proportion of adolescents and adults aware of their sickle cell trait status is broadening community‐based education regarding the potential for being a carrier for sickle cell disease (Taylor et al., 2014). Any community‐based efforts surrounding informing individuals of their status must concurrently include an emphasis on the broader dissemination of education for the public regarding sickle cell trait. Tandem roll‐out of these public health initiatives should occur to counter potential stigmatization and discrimination that has previously occurred related to sickle cell diagnoses (Markel, 1992).

Even among individuals with knowledge of their status, retention of information regarding sickle cell trait varies. A study of young adults with sickle cell disease or trait found that a web‐based intervention education program increased knowledge of sickle cell trait and disease, but did not affect intention or behavior to reduce their risk of having a child with sickle cell disease (Gallo et al., 2016). Another study of an in‐person educational intervention aimed at caregivers of children with sickle cell trait showed an increase in sickle cell trait knowledge that declined over time (Creary et al., 2017). Further research is necessary to understand the best approaches to providing individuals with sickle cell trait with the necessary information to make informed family planning decisions.

Our study is limited to children with SCA and sickle cell trait; additional research is necessary to understand the incidence, demographics, and geographic distribution of all sickle cell subtypes. Likewise, although this analysis focused on SCA and sickle cell trait, we acknowledge the importance of providing appropriate genetic counseling and family planning information to all individuals with hemoglobin variants (Pass et al., 2000).

In conclusion, the physical, mental, emotional, and social burden of SCA underscores the importance of education and outreach regarding sickle cell trait. This study emphasizes this importance of allocating additional resources for this education and outreach, given the 50‐fold greater incidence and substantially expanded geographic distribution of sickle cell trait compared to SCA. Further research is necessary to understand the most appropriate and impactful use of resources to increase the proportion of families and adults that are aware of their status.

## CONFLICT OF INTEREST

The authors have no conflict of interest. No financial disclosures were reported by the authors of this paper.
